# Perspectives on the utilization of resistance mechanisms from host and nonhost plants for durable protection of *Brassica* crops against Alternaria blight

**DOI:** 10.7717/peerj.7486

**Published:** 2019-09-26

**Authors:** Urooj Fatima, Priyadarshini Bhorali, Sudarshana Borah, Muthappa Senthil-Kumar

**Affiliations:** 1 National Institute of Plant Genome Research, New Delhi, Delhi, India; 2 Department of Agricultural Biotechnology, Assam Agricultural University, Jorhat, Assam, India

**Keywords:** Brassica, Alternaria Brassicae, Broad-spectrum defense, Blight-disease, Necrotrophic fungus, Nonhost resistance

## Abstract

**Background:**

*Alternaria brassicae*, the causal organism of Alternaria blight, is a necrotroph infecting crops of the *Brassicaceae* family at all growth stages. To circumvent this problem, several disease management strategies are being used in the field, and disease-resistant varieties have also been developed. However, no strategy has proven completely successful, owing to the high variability in virulence among *A. brassicae* isolates, which causes a diverse spectrum of symptoms. Nonhost resistance (NHR) is a robust and broad-spectrum defense mechanism available in plants, and the exploitation of gene pools from plant species that are nonhost to *A. brassicae* could serve as novel sources of resistance.

**Methodology:**

We searched the literature using key words relevant to this study in various search engines, such as PubMed, Web of Science, and Google Scholar, as well as certain journal websites. The literature was retrieved, sorted, and mined to extract data pertinent to the present review.

**Results:**

In this review, we have comprehensively covered the recent progress made in developing Alternaria blight resistance in *Brassica* crops by exploiting host germplasm. We also enumerate the potential NHR sources available for* A. brassicae* and the NHR layers possibly operating against this pathogen. In addition, we propose different strategies for identifying NHR-related genes from nonhost plants and testing their relevance in imparting broad-spectrum resistance when transferred to host plants.

**Conclusion:**

This review will help broaden the current knowledge base pertaining to the resistance sources available in host germplasm, the exploitation of NHR mechanisms, and their applications in protecting *Brassica* crops from Alternaria blight. The insights might also be applicable to a wider repertoire of plant pathogens.

## Introduction

Despite the considerable increase in the production and productivity of *Brassica* crops (*B. juncea*, *B. rapa*, *B. napus*), there remains a large discrepancy between yield potential and crop productivity in farmers’ fields owing to the plethora of diseases affecting these crops. Among these diseases, Alternaria blight, caused by the necrotrophic fungus *Alternaria brassicae*, is one of the most important diseases of oilseed brassicas throughout the world (Conn, Tewari & Awasthi, 1990; Saharan, Mehta & Meena, 2016). Two other species—*A. brassicicola* and *A. raphani*—are also involved in disease manifestation across the world. However, *A. brassicae* is highly infectious and more damaging to *Brassica* crops (Kadian & Saharan, 1983; Meena et al., 2010). *A. brassicae* can affect the host plant at all stages of growth, including seeds. Infected seeds and diseased plant debris are the sources of primary infection. However, under favorable environmental conditions, the pathogen spreads through airborne and soil-borne spores, resulting in secondary infection. *A. brassicae* has no known sexual stage and has been found to survive as mycelium or spores on decaying plant debris or as latent infection in seeds (Thomma, 2003). Under temperate conditions, the pathogen survives in the form of mycelia or conidia on the previous year’s crop debris and as chlamydospores or microsclerotia at cooler temperatures (Tsuneda & Skoropad, 1977;  Humperson-Jones & Maude 1982; Humpherson-Jones & Phelps, 1989). It also resides in alternative hosts such as susceptible weeds or perennial crops during the off season (Chupp & Sherf, 1960; Maude & Humpherson-Jones, 1980). The spores landing on the host surface as conidia adhere to the surface and penetrate the host mainly through the stomata after forming a germ tube, which then grows into the host epidermal cells through the formation of an appressorium, often triggered by host signaling mechanisms (Yang et al., 2005; Cho et al., 2006). The infection results in the appearance of severe disease symptoms, including numerous black spots covering the pods, leading to losses in seed yield of up to 60% (Kolte, 1985; Kolte, 2002; Meena et al., 2010). The severity of the disease differs among seasons and regions, as well as among crops within a region (Meena et al., 2012), which can be attributed to the variability among isolates of *A. brassicae* species. Different strategies have been adopted to control the disease, such as crop rotation practices and fungicide application; however, the devastating effect of this pathogen on yield loss is not effectively overcome. Among the different strategies, the most viable approach has been the utilization of resistant sources from host germplasm (Meena et al., 2010). The transfer of resistance from tolerant wild species of the Brassicaceae family to cultivated *Brassica* crops has been repeatedly attempted but has yielded limited success owing to variability in virulence among *A. brassicae* isolates.

Another potential strategy parallel to this could be the transfer of genes from nonhost plants conferring resistance to all the isolates of *A. brassicae*. Nonhost resistance (NHR) is the most durable form of plant immunity effective against all genetic variants of a pathogen (Heath, 2000; Mysore & Ryu, 2004; Senthil-Kumar & Mysore, 2013; Lee et al., 2017). NHR is multilayered and can be divided into two major types: the pre-invasion layer and the post-invasion layer (Nurnberger & Lipka, 2005; Lee et al., 2017; Fonseca & Mysore, 2018). The pre-invasion layer has both pre-formed and inducible defense mechanisms. Pre-formed defense involves the physical and chemical barriers that prevent fungal entry inside the plant (Lee et al., 2017; Fonseca & Mysore, 2018). In addition, the plant recognizes pathogen-associated molecular patterns (PAMPs) and elicitor molecules from fungal pathogens and induces defense responses against them. Meanwhile, the post-invasion layer mainly comprises inducible defense responses from the plant. In the post-invasion layer, the defense response culminates in a hypersensitive response (HR) and reactive oxygen species (ROS)-mediated cell death. These defense responses in the post-invasion layer can further restrict fungal growth inside the plant (Lee et al., 2017; Fonseca & Mysore, 2018). These NHR layers together offer durable resistance and can be utilized for developing disease-resistant crops. This calls for the identification of the genes involved in NHR and the elucidation of the NHR mechanisms against *A. brassicae*. Eventually, NHR-related genes can be transferred to host plants to confer durable resistance to *A. brassicae*. In this review, we provide insights on the resistance mechanisms operating in Alternaria blight-tolerant *Brassica* species and the underlying NHR mechanisms. We also propose the potential strategies for exploiting NHR mechanisms by using genomic tools for future protection of *Brassica* crops.

### Survey methodology

Several search engines, including PubMed, Web of Science advanced search, and Google Scholar, as well as specific journal websites, were used, and the search was performed based on key words specifically chosen for the topic. Literature was retrieved and sorted based on the relevance of the topic. The literature was then mined to extract data, and relevant articles published from 1960 to 2018 were used to support and elaborate the hypothesis of this review. Various citations from these articles were back-referred to obtain further detailed information. Together, the compiled information was processed by the authors to prepare the manuscript. Perspectives on the relevant concepts were incorporated based on the authors’ expertise in this field of research.

### Symptoms and epidemiology

Alternaria blight disease symptoms occur in the leaves, petiole, inflorescence, stem, pods, and seeds (Verma & Saharan, 1994). The initial symptoms of blight appear on the older leaves of the plant, as discrete pinpoint spots, which later enlarge and become surrounded by a distinct yellow halo with concentric rings (Kumar et al., 2014). These spots ultimately coalesce, forming large patches of chlorotic and necrotic foliar blight lesions, resulting in defoliation. The pathogen resides in the center of the lesions, which is surrounded by yellow halos, the zone created by the diffusion of fungal metabolites (Tewari, 1983; Agarwal et al., 1997). Symptoms produced by all three species of *Alternaria* are similar. However, the spots formed by *A. brassicae* are greyish in color (Kumar et al., 2014). On the stems, the spots are elongated, while they appear round and blackish on the pods. Infection on the leaves and pods reduces photosynthetic potential, which not only results in reduced seed development and weight but also in reduced oil content and quality and poor germination efficiency (Kolte, 1996; Meena et al., 2010). In severely infected plants, the entire pod gets covered with numerous black spots, ultimately causing significant yield loss due to premature shattering.

In field conditions, disease development and spread are generally favored under temperatures ranging from 12 °C to 27 °C and a relative humidity of 70%, while on pods, infection occurs at a daily temperature range of 20–30 °C, together with more than 9 h of sunshine and leaf wetness (Awasthi & Kolte, 1994). Frequent rains are favorable for disease initiation and spread on the leaves of *Brassica*, particularly during the rosette to flowering stages. Furthermore, the frequency of infection is the highest during the flowering and pod stages (Prasad, Saxena & Chandra, 2003). According to a report by Awasthi & Kolte (1994), the susceptibility of the plants increases with age 30 days after sowing. Plants less than 30 days old do not show any symptoms, but they are highly susceptible 60–90 days after sowing.

### Resistance mechanisms operating in wild and tolerant species of *Brassica*

Various studies have been carried out to analyze the effect of Alternaria blight infection on host plant physiology and metabolism, but reports on host–pathogen interactions at the genetic and molecular level are still limited. A recent study has indicated that *Arabidopsis* can be a used as an efficient model system for deciphering the genetic and molecular mechanisms involved during interaction with *A. brassicae* (Mandal, Rajarammohan & Kaur, 2018).

Studies on the mechanisms of resistance to Alternaria blight have implicated polygenes (Tripathi et al., 1980; Medhi, 1985; Zhang, Xu & Takahata, 1996; Krishnia, Saharan & Singh, 2000), while some studies have attributed it to dominant nuclear genes (Tripathi et al., 1978; Subudhi & Raut, 1994; Zhang et al., 1997). Thus far, different sources of resistance to *A. brassicae* have been identified from host germplasm, including wild and tolerant species of *Brassica*. The resistance in the host germplasm has different components, which mainly include structural components such as epicuticular waxes, as well as biochemical components like phenols and phytoalexins. Epicuticular waxes form a direct physical barrier in the plant, providing resistance to *Alternaria* (Conn, 1986; Conn & Tewari, 1989). High deposits of epicuticular wax, forming a protective hydrophobic coating on the leaf surface, reduce the adherence of inoculum, conidia germination, and germ tube formation in the plant (Saharan, 1992). Some Brassicaceae members, including *B. napus*, *B. carinata*, and *Sinapis alba*, have been reported to have higher epicuticular wax compared to *B. rapa* and *B. juncea*, and therefore, the former are less susceptible to Alternaria blight infection (Conn, Tewari & Hadziyev, 1984; Tewari, 1986). High quantities of epicuticular wax have been observed in the progeny of interspecific crosses between *B. napus* and *B. juncea* (Singh, Singh & Srivastava, 1999).

Alternaria blight-resistant varieties accumulate high amounts of biochemical compounds such as phenols. After infection, blight-tolerant species of *Brassica* such as *B. carinata* and *B. napus* have been observed to accumulate a higher amount of total phenols compared to the susceptible species *B. juncea* and *B. rapa* (Gupta et al., 1990; Gupta, Gupta & Kaushik, 1995; Gupta & Kaushik, 2002). Meanwhile, the levels of soluble and reducing sugars and soluble nitrogen have been found to be lower in resistant species. In another study, resistance to the disease was reported to be associated with increased levels of antioxidant enzymes of the phenolic pathway, such as polyphenol oxidase, peroxidase, and catalase (Singh et al., 2009). Furthermore, phytoalexin accumulation in response to pathogen infection and its role in disease resistance have been well studied in *Brassica*. Phytoalexins are low-molecular-weight antimicrobial compounds produced by plants after pathogen infection. Cultivars of both resistant (*B. napus*, *Camelina sativa*, *Eruca sativa*) and susceptible (*B. rapa*) species show the elicitation of phytoalexins (Tewari, Conn & Dahiya, 1987; Tewari, Conn & Dahiya, 1988; Conn, Tewari & Dahiya, 1988; Conn et al., 1991). The highly resistant *C. sativa* produces a large number of phytoalexins that are involved in regulating resistance to *A. brassicae* (Tewari, 1991). Rapid accumulation of phytoalexins in *C. sativa* after pathogen infection has been found to inhibit fungal growth on the leaf surface (Jejelowo, Conn & Tewari, 1991).

The *Alternaria* pathogen has also been found to secrete both host-specific and nonhost-specific toxic metabolites, enabling a wide range of infection symptoms (Nishimura & Kohmoto, 1983; Kohmoto, Otani & Tsuge, 1995; Agrios, 2005). These toxins have been shown to alter the permeability and functioning of the cell membrane and organelles, thereby inhibiting various physiological processes of the host plant (Mathur & Chand, 1991). Destruxin B, a host-specific *Alternaria* phytotoxin acts as a pathogenicity factor responsible for its aggressiveness and for the susceptibility of the host plant. The *Alternaria-* tolerant species *S. alba* detoxifies destruxin B, and this is followed by simultaneous phytoalexin formation and elicitation. Together, these processes constitute the resistance mechanism of *S. alba* against *A. brassicae* (Pedras & Smith, 1997; Pedras et al., 2001). Some compounds related to camalexin and 6-methoxycamalex have also been found to confer toxicity to *A. brassicae* (Dzurilla et al., 1998).

### Exploiting the resistance mechanisms from host germplasm for developing blight-resistant brassicas

Several attempts have been made to exploit resistance sources from the wild relatives of *Brassica* and transfer them into cultivated *Brassica* (Aneja & Agnihotri, 2013). Efforts have been made in screening blight-resistant cultivars; however, high levels of resistance to *A. brassicae* have not been observed among cultivated *Brassica* species. The species show varying degrees of resistance responses against Alternaria blight, and accordingly, *B. rapa* and *B. juncea* are classified as being more susceptible to the disease than *B. napus* and *B. carinata* (Skoropad & Tewari, 1977). Of late, studies aimed at identifying blight-tolerant oilseed *Brassica* germplasm have been increasing. Table S1 enlists the sources of resistance, including the related wild species or weedy varieties of *Brassica* against Alternaria blight. These related wild species have been found to harbor genes conferring resistance to Alternaria blight that can be used for resistance breeding (Ghose et al., 2008; Chatterjee, Mazumder & Basu, 2013).

In the past few decades, various conventional and modern approaches have been used to develop blight-resistant cultivars by exploiting resistance sources from host germplasm. Conventional approaches involve the screening of germplasm for blight-resistant plant materials, followed by the transfer of resistance traits from tolerant species into susceptible cultivars by hybridization combined with multiple crosses. Inter- and intraspecific crosses of *B. juncea* and *B. carinata* were established to understand the inheritance pattern of resistance to Alternaria blight (Krishnia, Saharan & Singh, 2000), which showed that dominant and additive genes govern the inheritance of resistance to Alternaria blight. Thus, a successful breeding method should utilize both the gene effects. However, there is a limitation in such breeding approaches owing to the low availability of suitable resistance sources/genes within the available germplasm of cultivated *Brassica* and insurmountable self-incompatibility barriers in combining the resistance traits from distantly related wild *Brassica* species. Modern biotechnological interventions involving tissue culture and genetic transformation approaches have also been established for the development of improved varieties. *In vitro* ovary culture and ovule culture have been attempted for transferring resistance traits from *S. alba* cv. Carine to *B. napus* cv. Brutor (Chevre et al., 1994) and *Erucastrum cardaminoides* to *B. oleracea* var. *alboglabra* (Mohanty et al., 2009). Intergeneric hybrids of *B. campestris* and *B. spinescens* have also been developed (Agnihotri et al., 1991). Moreover, the somatic hybridization technique has been applied for transferring the resistance traits from blight-tolerant species to susceptible ones, for example, *S. alba* to *B. napus* (Primard et al., 1988), *B. carinata* to *B. juncea* (Sharma & Singh, 1992), and *B. nigra* and *B. oleracea* (Jourdan & Salazar, 1993). Attempts have also been made for the introduction of somaclonal variations for the incorporation of disease resistance/tolerance to Alternaria blight through mutagenesis using gamma rays, ethyl methanesulfonate, and ethyl nitrosourea (Verma & Rai, 1980; Agnihotri et al., 2009). In addition, the defense response might be activated by treatment with biotic and abiotic agents for combating *A. brassicae* infection in oilseed brassicas. Vishwanath et al. (1999) showed that resistance in a susceptible cultivar of *B. juncea* against the extremely virulent *A. brassicae* isolate A and reasonably virulent isolate C, both from *B. carinata*, was induced after inoculating the plants with the avirulent *A. brassicae* isolate D. As another example, the application of β-aminobutyric acid on the leaves of *B. carinata* was found to induce resistance to *A. brassicae* (Chavan, Bhargava & Kamble, 2013). In future, the activation of the defense response without gene manipulation may act as a better substitute for the conventional use of fungicides. Unfortunately, till date, none of the conventional or modern biotechnological methods have been found to be feasible or stable in developing blight-resistant *Brassica* species. Under such circumstances, it is pragmatic to use novel sources of resistance from plant species that are not hosts to the pathogen. The transfer of genes from nonhost plants conferring resistance to the pathogen is a promising strategy in parallel to the current efforts towards the development of oilseed *Brassica* varieties resistant to Alternaria blight.

### Exploiting nonhost disease resistance mechanisms

NHR is expressed by all the members of a plant species to a particular pathogen (Nurnberger & Lipka, 2005). Exploiting NHR has been suggested as an option for developing broad-spectrum disease resistance in plants (Ellis, 2006; Lee et al., 2017). In recent years, considerable progress has been made in understanding the interaction of several host species and *A. brassicae*, but there are few and indirect reports on studies examining the NHR mechanisms involved during the interaction of *A. brassicae* with nonhost plants. Understanding the mechanisms and identifying the genes involved in NHR to *A. brassicae* may provide an opportunity to engineer transgenic *Brassica* plants that provide durable resistance to *A. brassicae*. For example, in recent years, some success in genetic transformation involving the transfer of resistance genes from nonhost plants into oilseed *Brassicas* has been reported. Mondal et al. (2003) found that the overexpression of tobacco chitinase conferred resistance to Alternaria leaf spot in transgenic *Brassica*, as demonstrated by the inhibitory effect of the transgenics on the hyphal growth of *A. brassicae*. They further reported the development of transgenic *B. juncea* (cv. RLM 198) constitutively expressing a class I basic glucanase of tomato, which arrested the hyphal growth of *A. brassicae* by 15–54% (Mondal et al., 2007). Moreover, the introduction of the chitin-binding lectin hevein from rubber plants into *B. juncea* led to resistance to *A. brassicae* (Kanrar et al., 2002). Such studies highlight the necessity to dissect the mechanisms and reveal the genes involved in NHR for the development of resistant cultivars.

**Table 1:** List of the potential nonhost plants towards *A. brassicae*.

**Sl. No.**	**Name of the nonhost plant**	**Name of the host plant intercropped with nonhost plant^a^**	**Criteria for categorization of nonhost plant**	**Information source**
1.	Chickpea (*Cicer arietinum* and *C. kabulium)*	*B. juncea, B. napus B. rapa*	Intercropping system	Gangasaran & Giri (1985), Tiwari et al. (1992), Ahlawat & Sharma (2002), Ahlawat, Gangaiah & Ompal (2005), Singh, Kumar & Singh (2010), Shekhawat et al. (2012), Jeromela et al. (2017)
2.	Lentils (*Lens culinaris*)	*B. juncea B. rapa*	Intercropping system	Gangasaran & Giri (1985), Tiwari et al. (1992), Shyam, David & Philip (2007), Shekhawat et al. (2012), Ao & Saud (2016), Jeromela et al. (2017)
3.	Barley (*Hordeum vulgare*)	*B. juncea B. rapa*	Intercropping system	Gangasaran & Giri (1985), Singh, Kumar & Singh (2010), Shekhawat et al. (2012)
4.	Wheat (*Triticum aestivum*)	*B. juncea B. napus B. rapa*	Intercropping system; Literature	McRoberts & Lennard (1996), Ahlawat & Sharma (2002), Singh, Kumar & Singh (2010), Shekhawat et al. (2012), Ebrahimi et al. (2016)
5.	Tomato (*Solanum Lycopersicum)*	–	Literature	McRoberts & Lennard (1996)
6.	Potato (*Solanum tuberosum*)	*B. juncea B. napus*	Intercropping system	Ahlawat & Sharma (2002), Singh, Kumar & Singh (2010), Shekhawat et al. (2012)
7.	Sugarcane (*Saccharum officinarum*)	*B. juncea, B. rapa, B. napus, B. campestris*	Intercropping system	Singh, Kothari & Tripathi (1986), Toor et al. (2000), Suman et al. (2006), Ahlawat & Sharma (2002), Singh, Kumar & Singh (2010)

**Notes.**

a Based on the information available on the plant wise knowledge bank database. ( http://www.plantwise.org/KnowledgeBank) it was ensured that the above listed nonhost plants are not reported to be infected by *A. brassicae*. The intercropping system was considered as a criteria for the selection of the nonhost plant, when both the host and nonhost plants are cultivated together for many decades and the pathogen get enough chance to infect the nonhost plant. Other nonhost plants are selected from the literature studies based on experimental evidence that suggest the plant as a nonhost for *A. brassicae*.

For this, the first step is to identify potential nonhost plants that can reveal robust and exploitable mechanisms for suitable transfer to a host plant for imparting broad-spectrum resistance. Besides, the nonhost plant should be distinct from the non-adapted plant, where, for example, the fungal spores fail to adhere, deposit, and remain on the leaf surface for germination (Niks & Rubiales, 2002). Here, we discuss some of the nonhost plants for *A. brassicae* based on the available literature and the intercropping systems followed in different parts of world. In many intercropping systems, species of the *Brassicaceae* family are cultivated with certain other plant species in the same field for many decades (Singh, Kumar & Singh, 2010; Shekhawat et al., 2012; Ao & Saud, 2016; Ebrahimi et al., 2016; Jeromela et al., 2017). In the same field, *A. brassicae* infects *Brassica* plants but cannot infect the other plant species (http://www.plantwise.org/KnowledgeBank). The Plantwise Knowledge Bank database (http://www.plantwise.org/KnowledgeBank) lists the plant species infected by *A. brassicae* in different parts of world. Through this database, we ensured that the identified nonhost plants have not been reported to be infected by *A. brassicae*. Hence, those species can act as potential nonhost plants against this pathogen. Curiously, in the course of evolution, the pathogen gets enough opportunities to infect these nonhost plants, but they are unable to overcome the NHR. A few examples of such nonhost plants, which are intercropped with host plants such as mustard (*B. juncea*), are chickpea (*Cicer arietinum* and *C. kabulium*), lentil (*Lens culinaris*), cowpea (*Vigna unguiculata*), barley (*Hordeum vulgare*), sugarcane (*Saccharum officinarum*), and potato (*Solanum tuberosum*) (Table 1). It would be interesting to determine the factors that dictate the failure of *A. brassicae* to infect these nonhost plants. The NHR of these plants to *A. brassicae* may involve multiple layers of defense that could be distinct for each plant species.

**Figure 1:**
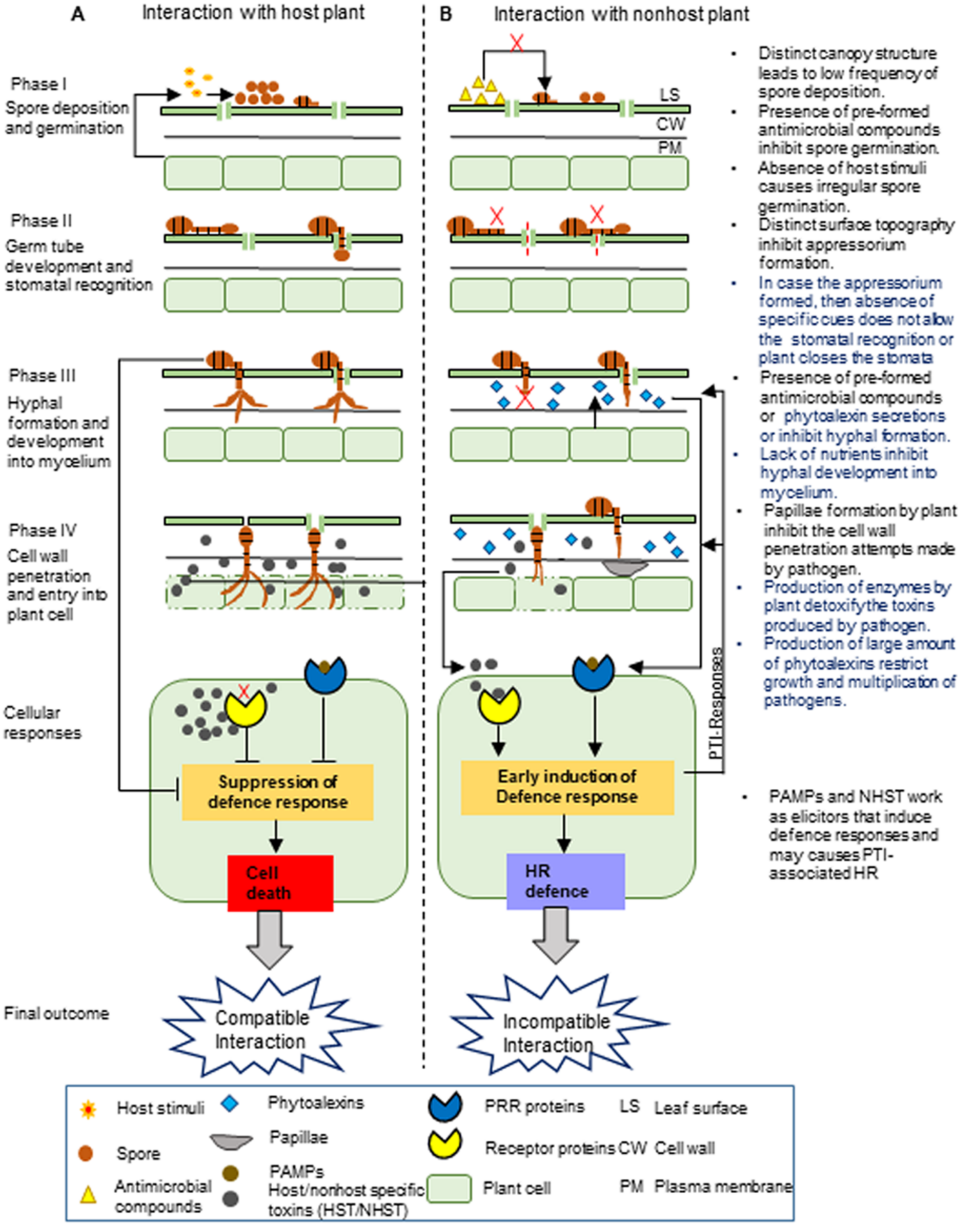
Proposed stages of nonhost resistance against *Alternaria brassicae*. This schematic representation depicts the possible events during the interaction of *Alternaria brassicae* with the host (A) and nonhost plants (B), which is divided into four different phases.The NHR towards* A. brassica* may involve the pre-formed and inducible defense barrier at different phases. I. The first phase involves the deposition and germination of *A. brassicae* spores on the leaf surface. Nonhost plants have a low frequency of spore deposition on the leaf surface due to the differences in their canopy structure compared to the host plant. Moreover, spore germination might be inhibited due to the presence of pre-formed antimicrobial compounds on the leaf surface and irregular germination of spores due to the absence of host stimuli. II. The second phase involves germ tube development from the spores and the recognition of the stomata. The difference in the leaf surface topography of the nonhost plant compared to the host plant may not allow the proper development of the germ tube into the appressorium. At the next level, even if the appressorium is formed, either stomata are closed by plant or the pathogen may fail to recognize the stomata due to the absence of specific cues, generally derived from the distinct composition and amount of cuticular waxes in the nonhost leaf, which might be entirely different in the case of the host leaf. III. The third phase involves fungal hyphal formation and its development into mycelium. The nonhost plant may inhibit hyphal formation and its development into mycelium by secreting inducible chemicals by pre-formed antimicrobial compounds or by limiting nutrient availability in the intercellular spaces. IV. The fourth phase involves the penetration of the fungal pathogen into the plant cell wall and its subsequent entry inside the plant cell. The nonhost plant may inhibit the penetration of the fungal hyphae into the plant cell wall by inducing the formation of papillae. The defense responses are induced by nonhost plants after recognizing PAMPs or elicitor molecules. This may lead to the production of enzymes and a large amount of phytoalexins by the nonhost plants, which detoxify the toxins produced by the fungal pathogen and restrict fungal growth and multiplication. **Note:**The text on the right in the figure indicates the NHR mechanisms, and the highlighted blue text indicates the most exploitable NHR mechanisms to develop durable disease-resistant *Brassica* crops.

### Possible NHR mechanisms in nonhost plants to *A. brassicae*

NHR to fungi is highly conserved among different plant species and seems to involve multiple layers of resistance (Heath, 2000). NHR mainly comprises pre-invasion and post-invasion defenses (Lee et al., 2017; Fonseca & Mysore, 2018). Based on the current understanding of the NHR mechanisms in other related nonhost plant–pathosystems such as *Arabidopsis* and powdery mildew fungus, barley and powdery mildew fungus, and *Medicago* and the rust fungal pathogen (Collins et al., 2003; Lipka et al., 2005; Jafary et al., 2008; Douchkov et al., 2014a; Douchkov et al., 2014b; Ishiga et al., 2015), we have proposed the possible NHR mechanisms in a nonhost plant against *A. brassicae* (Fig. 1). The NHR mechanisms operating at the pre-invasion level mainly involve both pre-formed and inducible defense responses. The germination of *A. brassicae* spores requires host stimuli, high humidity, and surface water to increase wetness on the leaf surface (Verma & Saharan, 1994). Pre-formed defenses may involve structural features such as the abundance of leaf trichomes, which reduces leaf wettability, as well as the lack of stimuli from the nonhost plant, which eventually causes irregular spore germination or a complete lack thereof (Niks & Rubiales, 2002). Pre-formed barriers also include chemical compounds present on the leaf surface, for example, antimicrobial enzymes and secondary metabolites, which might inhibit spore germination (Heath, 1997a; Heath, 1997b; Morrisey & Osbourn, 1999; Heath, 2000). The ability of *A. brassicae* spores to germinate on nonhost tomato and wheat plants was tested by McRoberts & Lennard (1996). They found that the spores were able to germinate with the same efficiency on the nonhost and host plants. These spores may have overcome the pre-formed chemical barriers, thereby being able to germinate on the leaf surface. Furthermore, the entry of germinated *A. brassicae* spores inside the plants is achieved either by targeting the stomata, which is a more predominant mode for entry, or directly through the cuticle (Changsri & Weber, 1960; Tsuneda & Skoropad, 1978). Stomatal recognition by a pathogen requires specific plant topographical cues, which allow germ tube development and eventually facilitate the penetration inside the plant (Hoch et al., 1987; Heath, 1997a; Staples & Hoch, 1997; Niks & Rubiales, 2002). A study on tomato and wheat plants showed that germ tube development of *A. brassicae* was not affected, but the pathogens failed to enter the stomata of the nonhost plants, unlike in the host plants (McRoberts & Lennard, 1996). It is possible that in the nonhost plants, stomata are not accurately recognized by the pathogen because the surface topography may significantly differ from that of the host leaf. The lack of stomatal recognition by the pathogen may lead to the development of the germ tube, albeit away from the location of the stomatal opening. This sort of disruption in the orientation of the germ tube may not allow the pathogen to enter inside the leaf tissue. Another pre-formed structural barrier includes cuticular waxes, which retard pathogen entry into the leaf tissues (Tewari & Skoropad, 1976; Skoropad & Tewari, 1977; Conn, 1986; Serrano et al., 2015). For example, barley has excessive cuticular wax, consisting of approximately 54% long-chain β-diketones, 20% hydroxylated β-diketones, and other hydrocarbons (Wettstein-Knowles & Netting, 1976; Simpson & Von Wettstein-Knowles, 1980). Together, these form thick cuticular layers that occlude the stomatal features involved in germ tube penetration inside the leaf tissue (Uppalapati et al., 2012). Besides, *Alternaria*, being a necrotrophic pathogen, sometimes directly enters the host plant through the cuticle by enzymatically digesting cutin waxes by means of cutinolytic enzymes (Tewari, 1986; Conn & Tewari, 1989). For a few tolerant host species, a high content of cuticular waxes acts as a physical barrier that provides resistance to *A. brassicae* (Tewari & Skoropad, 1976; Skoropad & Tewari, 1977; Conn & Tewari, 1989). We propound that nonhost plants may have appreciably high amounts of cuticular waxes, which may restrict pathogens from penetrating inside the plant. The penetration attempts by the pathogen on the nonhost plant may trigger inducible defense responses in the plant. The nonhost plant can induce stomatal closure, prevent the entry of *A. brassicae*, and mount an inducible chemical barrier involving the rapid formation of phytoalexins—antimicrobial compounds inhibiting hyphal development and differentiation (Grayer & Harborne, 1994; Mert-Turk, 2002; Iriti & Faoro, 2007). The differentiation of hyphae is important for penetration through intercellular spaces, and certain nutrients are essential for hyphal development and differentiation (Giri et al., 2013). Thus, in a nonhost plant, the lack of nutrients and presence of antimicrobial compounds in the apoplast may inhibit the development of hyphae into mycelium. Moreover, the defense responses also involve the formation of a structural barrier to arrest fungal growth and penetration in the cell wall (Underwood, 2007; Voigt, 2015). For example, *A. brassicae* fails to penetrate the cell wall in tomato and wheat plants, and callose-containing papilla formation is observed in the cell wall regions where the pathogen attempts to penetrate such plants(McRoberts & Lennard, 1996). The pathogen also produces nonhost-specific or general toxins that can damage the plant cells, which eventually leads to necrosis (Buchwaldt & Green, 1992; Bains, Tewari & Ayer, 1993; Parada et al., 2007). To avoid this, a nonhost plant may recognize these toxins and employ defense mechanisms to detoxify these toxins (Pedras et al., 2001). Some NHR mechanisms operate at the post-invasion level, which might restrict the pathogen from invading the epidermal cells in the nonhost plant. These defense responses mainly involve ROS accumulation and defense-induced cell death, which do not allow the mycelium to ramify through and between the cells. Eventually, fungal proliferation might be restricted by rapid HR (McRoberts & Lennard, 1996; Gilchrist, 1998).

### Understanding the molecular events in NHR to other *Alternaria* species

Defense responses are evoked in both host and nonhost infections; however, the NHR involves not only earlier induction but also a robust defense response compared to the host plant. Both *A. brassicicola* and *A. brassicae* affect almost the same cruciferous crops and generally exhibit similar symptoms (Saharan, Mehta & Meena, 2016; Meena et al., 2016). However, the literature indicates that *Arabidopsis* and *S. alba*, which belong to the same family (Brassicaceae) show NHR to *A. brassicicola* (Thomma et al., 1999; Pedras et al., 2001) but act as hosts for *A. brassicae* (Hansen & Earle, 1997; Sharma et al., 2002). A recent study showed *Arabidopsis* accession Gre-0 to be highly susceptible to *A. brassicae* (Mandal, Rajarammohan & Kaur, 2018). Therefore, understanding the NHR mechanisms in these plants against *A. brassicicola* will indirectly illustrate the resistance mechanisms operating during the interaction of *A. brassicae* with nonhost plants or absent during the interaction with these susceptible host plants. Hence, this section covers the molecular mechanisms of NHR to *A. brassicicola.*

In *A. thaliana* and *S. alba*, early and high-level induction of defense-related genes, namely, *pathogenesis-related-1* (*PR1*), β-1,3 *glucanase* (*PR2*), and *chitinase* (*PR3*), occurs compared to *B. juncea* after infection with *A. brassicae* or *A. brassicicola* (Narusaka et al., 2003; Van Wees et al., 2003; Ghose et al., 2008; Nayanakantha et al., 2016; Mandal, Rajarammohan & Kaur, 2018). Moreover, the expression of *PR3*, encoding chitinase, remained undetected in *B. juncea* after pathogen infection, but its expression was highly induced in *S. alba* (Nayanakantha et al., 2016). However, both *A. thaliana* and *S. alba* actively secrete chitinase enzymes, which hydrolyze the fungal cell wall and release chitin fragments (Narusaka et al., 2003; Chatterjee, Mazumder & Basu, 2013). These chitin fragments are recognized by some receptors that activate effective defense responses against the pathogens (Wan et al., 2008). This suggests that the defense-related genes are activated earlier in nonhost plants than in host plants as a part of the NHR strategy, and the chitinase capable of degrading fungal cell walls plays an important role in restricting pathogen growth at early stages of infection. Furthermore, the receptors involved in chitin recognition in *Arabidopsis*, e.g., CERK 1 or LysM receptor-like kinase (LysM-RLK) and phytosulfokine receptor kinase (PSK), were found to be induced after *A. brassicicola* infection (Wan et al., 2008; Loivamaki et al., 2010). Wan et al. (2008) also showed that a mutation in the *LysM-RLK* gene inhibits the induction of all chitin-responsive defense genes and compromises the NHR to *A. brassicicola*. Similarly, *Arabidopsis* homologs for RLK receptors were identified in *S. alba*, and their expression was highly induced after infection with *A. brassicicola*; the genes were downregulated in *B. juncea* (Ghose et al., 2008). This indicates that chitin digestion from the fungal cell wall and its perception by the plant may play an essential role during defense signaling in nonhost plants. The NHR response involves the stimulation of a signal transduction cascade after the perception of a pathogen by the plant cell, which initiates the activation of protein kinases and members of MAP kinases and subsequently leads to defense gene activation in nonhost plants (Lawrence et al., 2008). *MAPK6* expression was found to be highly induced for a longer duration in *S. alba* plants infected with *A. brassicicola*, but it was downregulated in *B. juncea* (Taj et al., 2010). These findings lead us to speculate that MAPK6 might be involved in imparting NHR and targeting many downstream components that are involved in effective defense response.

Salicylic acid (SA), jasmonic acid (JA), and abscisic acid (ABA) are the key modulators involved in phytohormone signaling during plant defense. JA-mediated signaling is predominantly involved in general defense against necrotrophic pathogens (Glazebrook, 2005). In *Arabidopsis*, JA-mediated activation of the defense response occurs against *A. brassicicola* (Thomma et al., 1998; Van Wees et al., 2003), while *S. alba* challenged with the same pathogen induces ABA-mediated and JA-mediated defense responses (Mazumder et al., 2013). In contrast, susceptible *B. juncea* plants induce SA-mediated defense signaling pathways (Thomma et al., 1998; Van Wees et al., 2003; Mazumder et al., 2013). It is likely that there is a significant amount of crosstalk between phytohormones and a convergence of two or more signaling pathways, which play a role in deciding whether disease progression will occur or the defense pathways will overcome the pathogen. Considering these studies together, we surmise that early signaling events in nonhost plants has a major role in imparting NHR to *A. brassicicola*.

### Possible strategies for developing durable Alternaria blight-resistant *Brassica* crops by using genomic tools

Based on a literature survey on intercropping systems, we have shortlisted the nonhost plants for *A. brassicae* (Table 1) that can be chosen for dissecting the NHR mechanisms. As discussed earlier, the NHR to *A. brassicae* may involve four different phases based on fungal invasion and penetration events (Fig. 1). Prior to any specific study, it is important to classify the nonhost plants based on these four phases by using pathological assays. This will provide a clear idea about whether the NHR operates at the pre-invasion or post-invasion level. Now, we outline the five major strategies for working towards the ultimate goal of developing durable Alternaria blight-resistant *Brassica* crops.

#### Inter or intra-specific introgression of candidate genes from nonhost plants to Brassica crops

The first strategy involves the identification of genes conferring NHR by utilizing conventional or molecular breeding approaches, followed by the introgression of candidate genes from nonhost plants to *Brassica* crops. This can be achieved in two ways. First, where possible, interspecific crosses of the host with a nonhost may be performed to transfer resistance traits from the nonhost plant to a host plant. The unwanted background in the progeny can be removed by crossing it with wild-type host plants and selecting for the targeted traits only. Then, the progeny can be screened for response to *A. brassicae*. Plants showing resistance to the pathogen can be subsequently subjected to QTL mapping to identify the genes involved in NHR. Once the genes are identified, they can be successfully introgressed into the desired varieties to provide resistance to *A. brassicae*. The major limitation with this approach, however, is that the progeny generally suffers from sterility and abnormal segregation, which can hinder the identification of genes involved in resistance. To our knowledge, the only available example of an interspecific cross made between a host and nonhost plant is that of *Lactuca* and *Bremia* (Jeuken et al., 2008).

**Figure 2:**
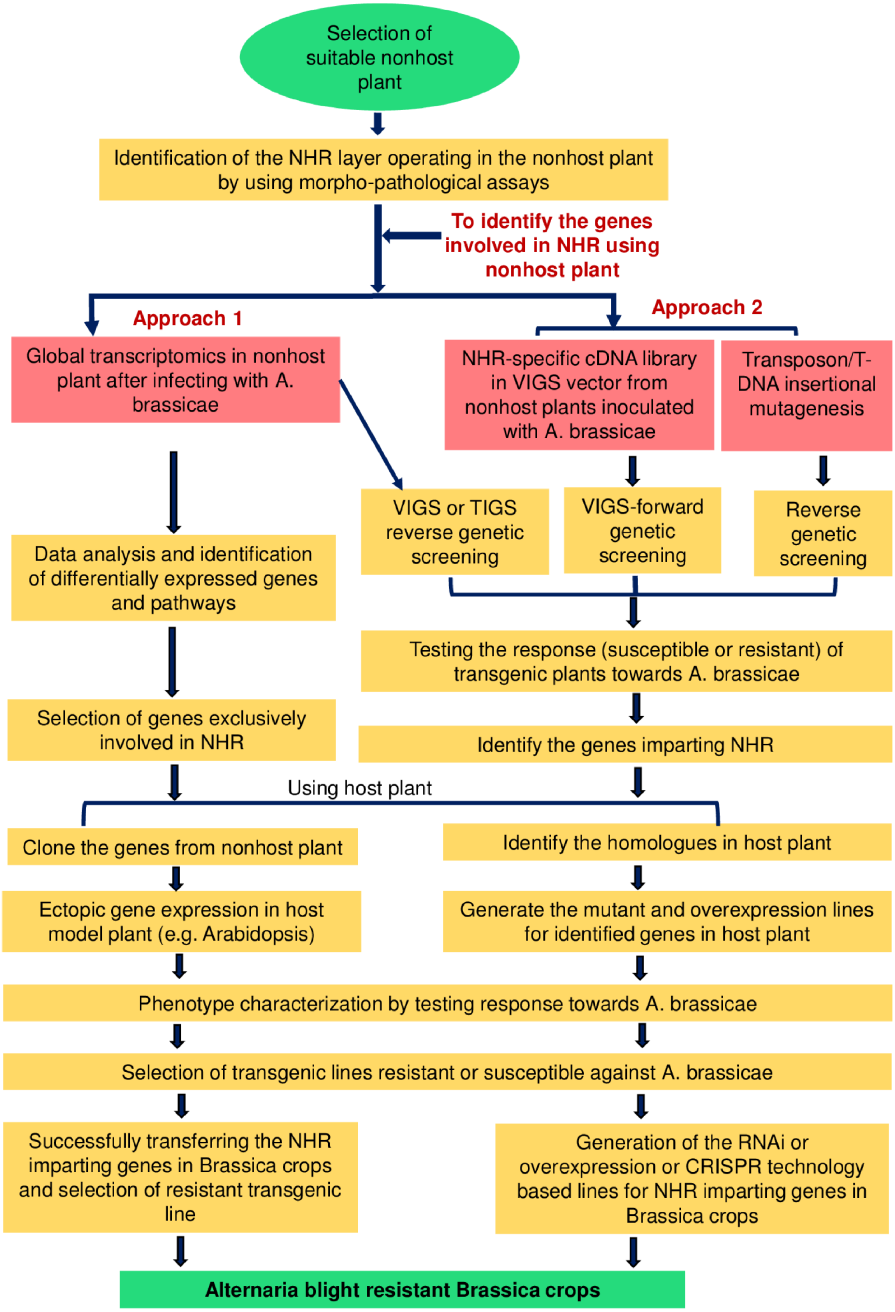
Schematic representation of the possible strategies for exploiting the NHR mechanisms to develop durable blight-resistant *Brassica* crops. The selection of a suitable nonhost plant is essential for exploiting NHR mechanisms. NHR may operate at two different layers, i.e., pre-invasive or post-invasive layers. Once the exploitable NHR layer is identified in the selected nonhost plant, several strategies can be suitably utilized for identifying the genes and pathways involved in the NHR mechanisms. The first approach involves global transcriptome profiling specific to* A. brassicae*-induced responses from the nonhost plant to identify genes playing a role in two different layers of NHR. The data can be enriched by using proper controls, e.g., non-adapted pathogens, and the genes exclusively involved in the NHR can be selected. The second approach involves the use of functional genomic tools, e.g., VIGS, TIGS, and T-DNA/transposon insertional mutagenesis, for identifying the genes.

The second approach is to cross two different varieties of nonhost plants (intraspecific cross: completely resistant genotype × moderately resistant genotype) that differ in the degree of resistance towards *A. brassicae*. This will allow the inheritance of NHR-specific QTL of the resistant donor into the moderately susceptible genotypes of the same species of the nonhost plant (Jafary et al., 2008; Niks & Marcel, 2009). The progeny obtained can be similarly studied as described above for identifying the genes involved in NHR and transferring them into target crops. However, if the nonhost plant does not have significant variation in the degree of resistance towards the pathogen, the inheritance of NHR-specific genes might be difficult (Fig. 2).

#### Microarray-based gene identification and transfer

The second strategy is to perform microarray analysis after infecting the nonhost plant (e.g., chickpea or barley) with *A. brassicae* and then identify the candidate genes from the list of upregulated or downregulated genes. If a homologous gene is identified, mutant or overexpression lines can be generated for the identified genes in the susceptible model plant. Alternatively, the candidate genes can be cloned from the nonhost plant and expressed in the susceptible model plant. These transgenic plants can then be tested for their response to *A. brassicae* infection. Finally, the genes imparting NHR to *A. brassicae* can be successfully transferred to *Brassica* crops.

#### Gene silencing

The third strategy to identify genes underlying NHR involves gene silencing in the nonhost plant. Virus-induced gene silencing (VIGS)-based forward genetic screening can be performed in nonhost plants that have established VIGS protocols and cDNA libraries to identify the genes involved in NHR to *A. brassicae*. For example, for wheat and barley, the *Barley stripe mosaic virus* (BSMV)-based vector and a well-developed VIGS protocol for gene silencing are available (Ma et al., 2012). Such a strategy will be useful in identifying important genes involved in NHR by screening a VIGS population for its response to *A. brassicae*. Furthermore, high-throughput transient expression and gene silencing methods, e.g., transient-induced gene silencing (TIGS), have been used in a nonhost plant such as barley. This technique is based on the transformation of leaf epidermal cells by the biolistic method (Douchkov et al., 2014a; Douchkov et al., 2014b). TIGS constructs may be made for the genes that are upregulated in the nonhost plant after *A. brassicae* infection. These TIGS constructs can be bombarded into leaf epidermal cells of the nonhost plant to induce gene silencing. After bombardment, leaf discs can be infected with *A. brassicae* and screened for the response. TIGS will allow the identification of potentially important candidate genes involved in NHR to *A. brassicae*. The genes identified using any of these functional genomic methods can be successfully transferred to *Brassica* crops to ensure resistance to *A. brassicae*.

#### Identification and use of model plants as genetic resources

The fourth strategy is to work with model plants that have well-developed molecular and genetic resources and a large population of mutants as nonhosts for *A. brassicae*. For example, *Brachypodium distachyon* can be directly utilized for identifying genes and dissecting the pathways involved in NHR (An et al., 2016). If the mutants are not readily available, then with the help of well-developed genetic tools, transposon or T-DNA insertional mutagenesis can be attempted in the model nonhost plant. The mutant population can be screened for the response to *A. brassicae* to identify the candidate genes involved in NHR. These candidate genes can then be successfully transferred to *Brassica* crops for durable resistance to *A. brassicae*. *Arabidopsis* is a well-established, easy-to-manipulate model plant with ample genetic resources. Another approach can be screening the response of all available *Arabidopsis* accessions to *A. brassicae*. This may help identify *Arabidopsis* accessions imparting resistance to *A. brassicae* (Rajarammohan et al., 2017; Mandal, Rajarammohan & Kaur, 2018).

#### Literature mining and targeted functional genomics

The fifth strategy is solely based on literature mining and targeted functional genomics to dissect the different layers of NHR involved in pre- and post-invasive defense responses. The available literature provides hints about possible candidates specifically involved in pre- and post-invasive defenses. This will provide the opportunity to directly start studying these candidates for their role in NHR. Another possibility is that the post-invasive defenses may play a more important role in restricting the fungal pathogen rather than pre-invasive defenses against *A. brassicae*, which can be determined by performing pathological assays. In such cases, we can use mutants with mutations in the genes important for pre-invasion defense to identify the genes involved in post-invasion defenses. For example, the mutant of the barley gene *Ror1*, a homolog of the *Arabidopsis* gene *Pen1*, is important for pre-invasive defense against many nonhost pathogens, including rust pathogens (Collins et al., 2003; Trujillo et al., 2004). This mutant line can be used for identifying genes involved in the post-invasion defense response. The response of a *ror1* mutant line should be tested towards *A. brassicae* to ascertain whether this gene is involved in pre-invasive defense against this pathogen. Subsequently, the NHR-specific candidate genes can be identified by transcriptomic analysis in wild-type barley after *A. brassicae* infection. Gene silencing approaches can be applied to silence the candidate genes in the *ror1* mutant line for identifying their role in post-invasion immunity by screening them against *A. brassicae.* Furthermore, validation experiments can be carried out by constructing double mutants of candidate genes and *Ror1* in barley. The double mutants compromising NHR and showing a higher degree of susceptibility to *A. brassicae* compared to the single mutant *ror1* can be likely candidates that contribute to NHR at the post-invasion level. The important genes identified can be further transferred, or their homologs can be overexpressed in the susceptible *Brassica* crops to test their resistance to *A. brassicae*.

### Conclusion and future perspectives

Selection from within the existing germplasm and the utilization of these sources to incorporate resistance in cultivated varieties through conventional breeding techniques have embodied the basic approaches for generating disease-resistant varieties. However, the exploitation of resistance mechanisms from host germplasm sources has shown limited success in the development of disease-resistant crop varieties. Meanwhile, when genes are introduced from related genera or families, the durability of resistance might be questionable, particularly when a single gene regulating one pathway for defense is employed. Therefore, it is essential to conduct parallel research based on the exploitation of NHR sources or genes against the pathogen over the course of developing varieties resistant to Alternaria blight. Compared to other forms of resistance, NHR governs defense responses to a broad range of pathogens. It is a stable, durable, and robust form of resistance that relies on multiple defense components. Sustainable and broad-spectrum resistance under field conditions makes NHR a promising resource for crop improvement. We speculate that the strategies proposed for exploiting NHR by applying the latest molecular biology techniques involving transcriptomic approaches and genomic tools such as VIGS, TIGS, and mutagenesis will be useful in identifying novel sources of NHR and developing *Brassica* crops with durable disease resistance. The utilization of model plants can be pivotal in identifying the genes and deciphering the pathways operational during NHR. A model plant can serve as a promising tool to enable plant breeders to transfer the knowledge obtained from it into crop plants for developing disease-resistant crops. However, the utilization of NHR sources for effective crop improvement is beset by several limitations, the major one being that one sole NHR mechanism cannot be exploited to combat all the pathogens of a crop. NHR is a more complex form of defense due to the involvement of multiple pathways; hence, it is less feasible in crop breeding applications. Furthermore, pathogens having a rapid evolutionary rate in nature tend to overcome the NHR mechanisms operating in the nonhost plants.

##  Supplemental Information

10.7717/peerj.7486/supp-1 Supplemental Information 1 Resistance sources reported in *Brassica* species and its utilization for management of Alternaria blight *This information is based on the results obtained from experiments on testing the response of *A. brassicae* towards above mentioned host plant species.

## Additional Information and Declarations

### Data Availability

The following information was supplied regarding data availability: This is a review article. Table S1 provides information on resistant sources. No other raw data are available for the article.
