# Supplementary material for: Perspectives on the utilization of resistance mechanisms from host and nonhost plants for durable protection of Brassica crops against Alternaria blight
Source: PeerJ. 2019 Sep 26;7:e7486. doi: 10.7717/peerj.7486 (PMC6766370; doi:10.7717/peerj.7486)
Supplement: Supplemental Information 1 — *This information is based on the results obtained from experiments on testing the response of A. brassicae towards above mentioned host plant species. [file peerj-07-7486-s001.docx]

**Supplementary table 1. Resistance sources reported in *Brassica* species and its utilization for management of Alternaria blight**

| **Sl. No.** | **Name of the host plant** | **Source of resistance in the germplasm** | **Mechanism of resistance** | **Extent of resistance offered to *A. brassicae**** | **References** |
| --- | --- | --- | --- | --- | --- |
| 1. | *Brassica. juncea* | Divya  RC781  exotic *B. juncea* strains, EC-399296, EC- 399299, EC-399301 and EC-399313  Kranti, PR 8988 and PR 9024 | Unknown  Unknown  Unknown  Unknown | Mild  Mild  Mild  low | Kolte *et al.*, 2000;  Tripathi *et al*., 1980  Kumar, 2008; Kolte *et al*., 2008 |
| 2. | *B.rapa* | *B. rapa var.* Yellow sarson, PYS6, BINA1,2  *B. rapa* rapifera | Unknown  Increased production of phytoalexins | Mild  Mild | Kolte, 1987;  Rahman *et al*.,  1987;  Conn *et al*.,  1988 |
| 3. | *B.carinata* | HC1,HC2,  EC25381, PCC2 | High level of cuticular wax | High  Mild | Kumar and  Saharan, 2002  Bhowmik and  Munde, 1987 |
| 4. | *B. napus* | Tower, HNS3  EC-338986-2 and EC- 338996-1; EC 339000 and EC 338997  GS-05-1 | High level of cuticular wax | High  High  High | Tiwari,1986  Kumar and Kumar, 1989  Kolte *et al*, 2008  AICRP, 2011  Kumar *et al*., 2014 |
| 5. | *Sinapis alba* | - | High level of cuticular wax  Detoxification of phytotoxin  Increased production of phytoalexin | High | Hansen and Earle, 1997 |
| 6. | *Camelina sativa* | - | High deposits of epicuticular wax  Increased production of phytoalexins i.e Camalexins | High | Tewari and Conn,1993; Browne *et al*.,1999 |
| 7. | *Capsella bursa-pastoris* | - | High deposits of epicuticular wax  Increased production of phytoalexins | High | Tewari and Conn,1993 |
| 8. | *Crantz* | - | High deposits of epicuticular wax  Increased production of phytoalexins | High | Tewari and Conn,1993 |
| 9. | *Neslia paniculata* | - | High deposits of epicuticular wax  Increased production of phytoalexins | High | Tewari and Conn,1993 |
| 10. | *Taramira* | - | High deposits of epicuticular wax  Increased production of phytoalexins | High | Tewari and Conn,1993 |
| 11. | *B. maurorum* | - | High deposits of epicuticular wax  Increased production of phytoalexins | High | Chrungu *et al.*,  1999 |
| 12. | *B. desnottesii* | - | High deposits of epicuticular wax  Increased production of phytoalexins | High | Sharma *et al*.,  2002 |
| 13. | *Coincya pseuderucastrum* | - | High deposits of epicuticular wax  Increased production of phytoalexins | High | Sharma *et al*.,  2002 |
| 14. | *Diplotaxis berthautii* | - | High deposits of epicuticular wax  Increased production of phytoalexins | High | Sharma *et al*.,  2002 |
| 15. | *D. catholica* | - | High deposits of epicuticular wax  Increased production of phytoalexins | High | Sharma *et al*.,  2002 |
| 16. | *D. cretacea* | - | High deposits of epicuticular wax  Increased production of phytoalexins | High | Sharma *et al*.,  2002 |
| 17. | *D. erucoides* | - | High deposits of epicuticular wax  Increased production of phytoalexins | High | Sharma *et al.,*  2002 |
| 18. | *Erucastrum gallicum* | - | High deposits of epicuticular wax  Increased production of phytoalexins | High | Sma et al., 20 Sharma *et al*.,  2002 |

*This information is based on the results obtained from experiments on testing the response of *A. brassicae* towards above mentioned host plant species.

**References:**

AICRP, 2011. *All India Coordinated Research Project* on Rapeseed-Mustard.

Chrungu B, Verma N, Mohanty A, Pradhan A, Shivanna KR 1999. Production and characterization of interspecific hybrids between Brassica maurorum and crop brassicas. *Theor Appl Genet*, 98: 608-613.

Conn KL, Tewari JP, Dahiya JS 1988 Resistance to *Alternaria brassicae* and phytoalexin-elicitation in rapeseed and other crucifers. *Plant Sci*, 55:21-25.

Hansen LN, Earle ED 1997. Somatic hybrids between *Brassica oleracea* and *Sinapis alba* L. with resistance to *Alternaria brassicae* (Berk.) Sacc. *Theor Appl Genet*., 94: 1078-1085.

Kolte SJ 1987 Important disease of rapeseed and mustard in india. Present research progress and future research needs. In: Proceedings of IDRC (Canada) 3^rd^ Oil Crop Network Workshop, Addis Ababa, 6: 91-106

Kolte SJ, Awasthi RP, Vishwanath 2000. Divya mustard: a useful source to create Alternaria black spot tolerant dwarf varieties of oilseed brassicas. *Pl Varieties Seeds,* 13: 107-111.

Kolte SJ, Nashaat NI, Kumar A, Awasthi RP, Chauhan JS 2008 Towards improving the genetic base of rapeseed-mustard through an Indo-UK research collaboration. *Indian J Plant Gen Res*, 21:132-137.

Kumar PR, Kumar P 1989 Genetic improvement of rapeseed mustard, achievements, critical gaps and future priorities. *J Oilseed Res*, 6:211-219

Kumar S, and Saharan GS 2002 Sources of multiple disease resistance in *Brassicae* spp. *J Mycol Plant Pathol*, 32:184-188.

Kumar B 2008. Assessment of slow blighting (*Alternaria brassicae*) resistance and yield in Indian mustard (*Brassica juncea* (L.) Czern. & Coss.) *Indian Phytopathol*, 61(2): 171-183.

Kumar D, Maurya N, Bharati YK, Kumar A, Kumar K, Srivastava K, Chand G, Singh SK, Mishra RK, Kumar A 2014. Alternaria blight of oilseed Brassicas: a comprehensive review. *African J Microbiol Res*, 8 (30): 2816-2829.

Munde PN, Bhowmik TP 1985 A source of morphological resistance to leaf blight disease of rapeseed and mustard caused by *Alternaria brassicae. Curr Sci,* 54:514-515.

Rahman A, Das ML, Howlider MAR, Mansur MA 1987 Promising mutants in Brassicae campestris. *Mutat Breed Newsl*, 29:14-15

Sharma G, Kumar DV, Haque A, Bhat SR, Shyam P, Chopra VL 2002. Brassica coenospecies: a rich reservoir for genetic resistance to leaf spot caused by *Alternaria brassicae*. *Euphytica*, 125:411-419.

Tewari JP 1986. Subcuticular growth of *Alternaria brassicae* in rapeseed. *Can J Bot*, 64: 1227-1231.

Tewari JP and Conn KL 1993. Reaction of some wild crucifers to *Alternaria brassicae*. *Bull OILBSROP*, 16: 53-58.

Tripathi NN, Kaushik CD, Yadav TP, Yadav AK 1980. Alternaria leaf spot resistance in raya. Haryana Agricultural University Journal of Research, 10: 166-168.
